# Effect of personality traits on matching dolls and their makers

**DOI:** 10.3389/fpsyg.2022.1045226

**Published:** 2023-01-20

**Authors:** Miki Uetsuki, Misako Kimura

**Affiliations:** ^1^Department of Community Studies, Aoyama Gakuin University, Kanagawa, Japan; ^2^Department of Contemporary Liberal Arts, Aoyama Gakuin Women’s Junior College, Tokyo, Japan; ^3^Department of Childcare, Hakodate Junior College, Hokkaido, Japan

**Keywords:** doll-maker resemblance, object-maker resemblance, dog-owner resemblance, personality traits, face recognition

## Abstract

Studies have shown that possessions such as cars and dogs resemble their owners, and products such as dolls resemble their makers even when students make them. We conducted three experiments to examine which part of the dolls resembled their makers. The results demonstrated that people match dolls to their makers when their eye regions were masked (Experiment 1), and the matching is possible even with the back views of the dolls (Experiment 2). These results may indicate people match dolls to their makers based on resemblances other than faces. Experiment 3 demonstrated that no effect of resemblance in personality traits was observed when dolls’ faces were visible. However, the resemblance of personality traits assumed by the dolls and their makers play an important role in the matching judgment when dolls’ faces were invisible (because of back views).

## 1. Introduction

Several studies have demonstrated that couples resemble each other (Zajonc et al., 1987; Hinsz, 1989), and this tendency is not limited to heterosexual couples (Abel and Kruger, 2011). Additionally, Keller et al. (1996) demonstrated that married couples showed resemblances not only in physical but also in psychological traits. Little et al. (2006) and Wong et al. (2018) also suggested that couples resemble in their perceived personality traits. Pets, like human couples, also resemble their owners. Coren (1999), Payne and Jaffe (2005), Roy and Christenfeld (2004, 2005), Nakajima et al. (2009), and Nakajima (2013) demonstrated that dogs resemble their owners. People also resemble their personal belongings, including inanimate objects. Alpers and Gerdes (2006) and Stieger and Voracek (2014) demonstrated that cars resemble their owners. There are several reasons why couples and dogs and owners look alike. However, the “mere exposure effect” and the “self seeks like” algorithm comprise the leading theories. The “mere exposure effect” stipulates that people tend to prefer familiar things (Zajonc, 1968; Moreland and Zajonc, 1982; Hinsz, 1989). Thus, humans who are familiar with their own faces in the mirror are likely to choose people with similar faces as their spouses and prefer dogs with similar faces. In contrast, “self seeks like” is an evolutionarily shaped algorithm that is characterized by assortative mating (Alvarez and Jaffe, 2004; Payne and Jaffe, 2005). Furthermore, if facial features are determined by genetic factors, assortative mating should be detected based on facial visual cues. Thus, a partner with a similar face to ours was selected. Moreover, “self seeks like” is also applied in situations where no reproductive purpose is involved (Alvarez and Jaffe, 2004). Particularly, Payne and Jaffe (2005) have suggested that people choose their pets by applying this algorithm.

These studies on couples, pets, and cars were concerned with resemblance when choosing something. However, a resemblance was also found when making objects. Uetsuki and Kimura (2022) demonstrated the resemblance between dolls and their makers in the case of cloth dolls (puppets). Doll collectors can customize dolls with physical traits such as skin, eyes, and wigs (Ignacio and Cupchik, 2020) and psychological traits such as warriors. Furthermore, Ignacio and Cupchik (2020) underscored that dolls represent the externalization of the doll collectors’ inner worlds. Particularly, these worlds are filled with fantastic stories and imaginative characters, referred to as “world-building” by Heljakka and Harviainen (2019). Thus, dolls can embody part of collectors’ personalities (Heljakka, 2012). Unsurprisingly, the study found that doll makers also project themselves onto dolls, resulting in a resemblance between doll-makers and dolls.

This study examines which part of the dolls resembled their makers. Regarding dogs and their owners, Nakajima (2013) examined which part of the face is critical for the judgment of dog-owner resemblance using a questionnaire with a matching task using the photographs of the dogs and their owners. The results suggest that the dogs and their owners resemble each other in the eye region. This is consistent with the claim that the upper face, including the eye region, is also used as a clue to judge kin recognition (Dal Martello and Maloney, 2006), and the information of eye regions can easily retain memories (McKelvie, 1976). Thus, Experiment 1 examined whether the dolls and their makers resembled each other in the eye regions.

## 2. Experiment 1

Experiment 1 examined whether doll-maker matching was possible when eye regions were masked. A decline in matching performance when the eye regions are masked, as in Nakajima (2013), would suggest that their eye regions are similar, and it plays an important role in doll-maker matching. However, if the participants can match the dolls and their makers even when the eye regions are masked, then, this would suggest that people do not match dolls and their makers based solely on their resemblance of the eye regions.

### 2.1. Methods

#### 2.1.1. Participants

One hundred forty-four students (72 females, 71 males and 1 other; average 18.63 ± 0.85 years old; hereinafter, ± shows the standard deviation) at a university in Kanagawa participated in the experiment voluntarily. The sample size was determined based on power analysis [the calculated sample size was 88, when effect size (*w*) = 0.3, *α* error probability = 0.05, power (1 − *β* error probability) = 0.8, and *df* = 1]. The photo judgment task was inoffensive, and written agreements were obtained from all participants.

#### 2.1.2. Ethics statement

This study was conducted following the recommendations of the Provisions of Experiments, Ethics Committee of Hakodate Junior College, or of the Ethics Regulations Concerning Research Involving Human Subjects of Aoyama Gakuin University. The students who made the dolls permitted the use of photos in the experiment. The study protocol followed the Declaration of Helsinki and was approved by the Ethics Committee of Hakodate Junior College or Aoyama Gakuin University.

#### 2.1.3. Stimuli

**Figure 1:**
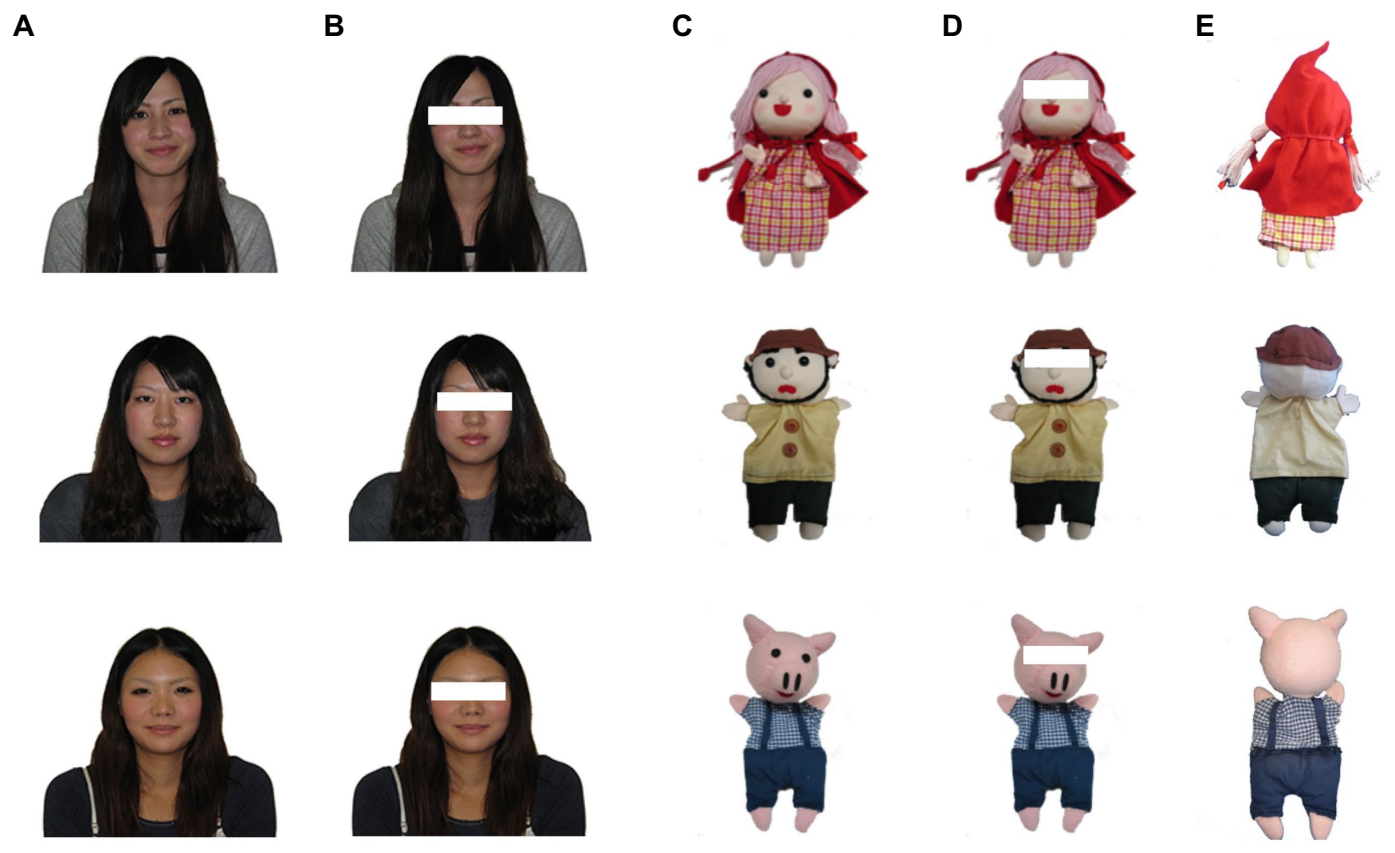
Examples of stimuli used in this study. The upper doll is a little girl with a red hood, and the middle doll is a hunter from “Little Red Riding Hood.” The lower doll is a pig from “The Three Little Pigs.” We used the following types of stimuli; **(A)** a maker with no mask, **(B)** a maker having the eye regions masked, **(C)** a doll with no mask, **(D)** a doll having the eye regions masked, and **(E)** back view of the doll. In Experiment 1, we used stimuli **(B,D)**.

The stimuli used in this experiment were identical to those used in Uetsuki and Kimura (2022). Photos of 30 college students each of whom had made a doll for a puppet show in the class “Teaching Methods” for childminders and kindergarten teachers, and the photos of the dolls were used as stimuli (Figure 1). They were all amateur doll-makers who made dolls for class credits. The backgrounds of the photos were omitted using a graphics software (Adobe Photoshop).

**Figure 2:**
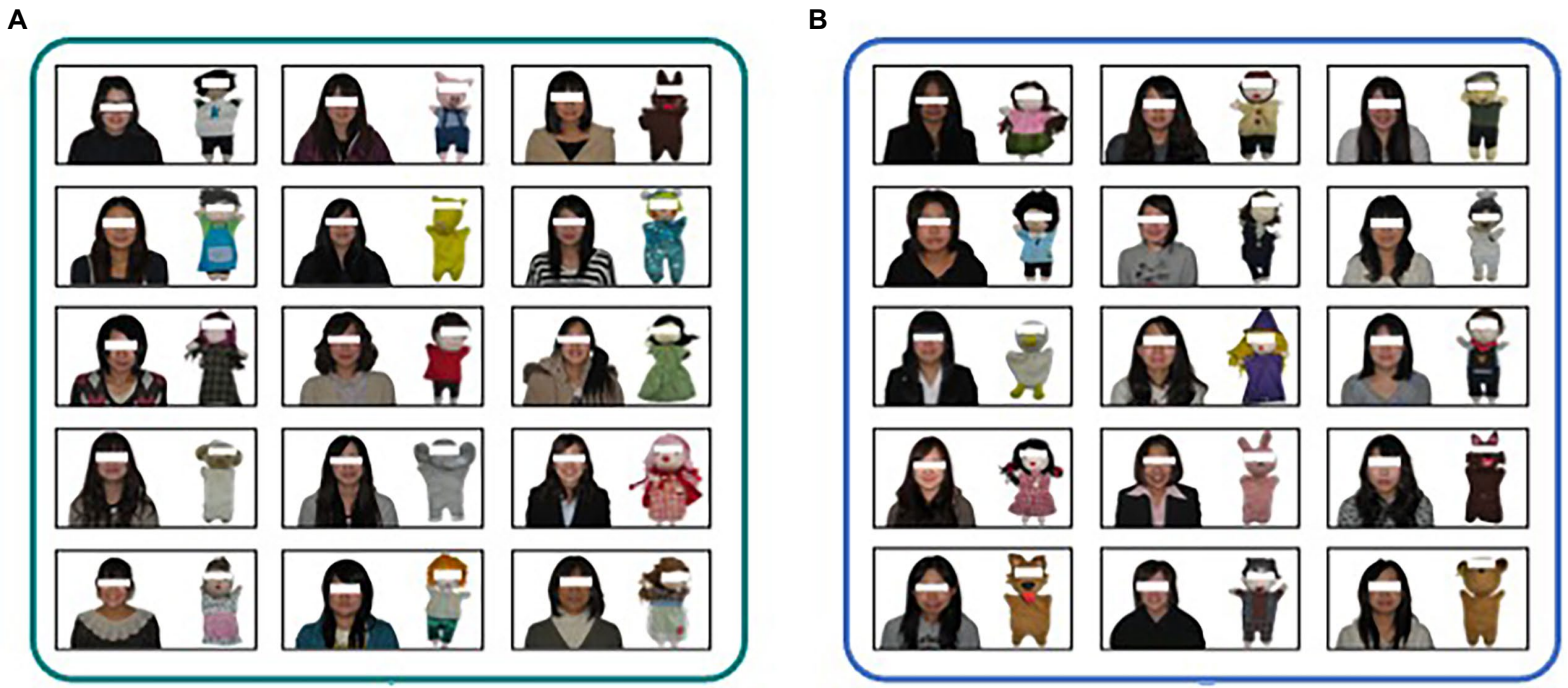
A sample of stimuli in Experiment 1. The participants were required to choose the matching doll-maker pairs, **(A)** or **(B)**.

Photos of the 30 dolls and their makers were divided into two sets, as in Uetsuki and Kimura (2022). One of the two sets comprised the “matching” pairs that had 15 correct (real) doll-maker pairs. The other set contained the “mismatching” pairs that had 15 incorrect pairs, made by swapping the dolls’ photos within the 15 makers. The two sets were used for both the matching and mismatching pairs. Figure 2 shows an example of our questionnaires. Photographs of the pairs were allocated 3 × 5 in each group. Squares with different colors surrounded each matching and mismatching group, and were labeled A or B. Consistent with Uetsuki and Kimura (2022), the placements of the stimulus photos and combinations of mismatching pairs were changed. Thus, 16 questionnaire patterns were used in this study. The questionnaire was printed on a sheet of A3 (420 mm × 297 mm), and the size of the photos of the students was about 2.6 cm × 2.6 cm, and that of dolls was about 1.8 cm × 2.6 cm. Here, the eye regions of students and dolls were masked with white squares (about 0.8 cm × 0.2 cm for students, 0.7 cm × 0.2 cm for dolls) as shown in Figures 1B,D. All the photos were printed in color.

#### 2.1.4. Procedures

One of the 16 questionnaire patterns was distributed among the participants. As the questionnaires were randomly and blindly distributed to the participants, the number of participants varied slightly between the 16 versions of the questionnaire. Each participant listened to and read the instructions. Discussions with other participants were strictly prohibited during the experiment. The participants had to fill in their ages and genders in the questionnaire. Subsequently, the participants were required to choose the matching doll-maker pairs, A or B. They answered by circling either A or B on the questionnaire in a two-alternative forced choice (2AFC). The questionnaire comprised only one question (i.e., a guessing task on the dolls and their makers) and took approximately 5 min including instructions. The dependent variable was the correct rate informed by the proportion of participants who chose the matching (i.e., correct) pair group out of the total number of participants. The procedure was the same as that used by Uetsuki and Kimura (2022).

### 2.2. Results and discussions

The rate of choosing the matching doll-maker pairs by the participants was 59.72% (86 participants chose matching pairs while 58 participants chose mismatching pairs) when the eye regions of the dolls and makers were masked. A chi-square test showed that the numbers of matching and mismatching choices differed significantly [*χ*^2^ (1) = 5.44, *φ* = 0.19, *p* < 0.05]. The results show that the participants could match the dolls to their makers above a chance level (50%) when the eye regions were masked. These findings are inconsistent with the matching dog-owner pairs in Nakajima (2013).

Uetsuki and Kimura (2022) used the same stimuli without a mask (Figures 1A,C) which yielded a correct rate of 64.71%. The results indicated that the correct rate was slightly lower when the eye regions were masked as opposed to when there was no mask. Consequently, this finding could suggest that the resemblance of the eye regions partially affects the judgment of the dolls and their makers. In contrast, the results demonstrated that people match the dolls to their makers when the eye regions were masked, suggesting that people do not match dolls and their makers solely based on their resemblance to the eye regions. Therefore, these findings propose that the dolls and their makers have similarities in the eye regions and areas other than the eyes. In any case, the following question arises: how do participants match dolls with their makers when the eye regions are lacking? Experiment 2 examines this question.

## 3. Experiment 2

This experiment examined whether people could match the dolls and their makers from the back views of the dolls. Thus, we used the back views of the dolls as stimuli to examine whether the entire face (including elements such as eyes or mouth, and the arrangement of the elements) could be an important clue for matching. If people match dolls and their makers based on their faces, then the matching performance might be at a chance level. However, if people match dolls and their makers based on information except for faces, then the matching performance might be above a chance level.

### 3.1. Methods

#### 3.1.1. Participants

Ninety-four university students (78 females and 16 males; average 20.13 ± 0.89 years old) in Kanagawa participated in the experiment voluntarily. The sample size was determined based on the results of Experiment 1. The photo judgment task was inoffensive, and written agreements were obtained from all participants.

#### 3.1.2. Stimuli

Only 11 out of the 30 dolls used in Experiment 1 could be photographed with their back views (Figure 1E), because they were actually used in puppet shows, and some of them were worn out or lost. Here, photos of five dolls that were not used in Experiment 1 were added as 11 dolls were not enough as stimuli. However, there was only one photograph of their makers. Thus, we had a total of 12 matching doll-makers pairs. We randomly selected 8 out of 12 doll-maker pairs (they were used as “matching” doll-maker pairs). We also used the photos of eight students consisting of “mismatching” doll-maker pairs. We could not take the photos of the makers’ backs as the doll-makers had graduate from college; therefore, the front views of their photographs were used (Figure 1A). Photos of pairs were allocated 4 × 2 in each group. We created eight patterns of questionnaires with different positions of the photos and combinations of mismatching pairs. The size of the students’ photos was about 3.6 cm × 3.6 cm, and that of the dolls’ photos was about 2.7 cm × 3.6 cm. The other parameters were the same as those in Experiment 1.

#### 3.1.3. Procedures

The questionnaire was distributed to the participants who were asked to choose the matching doll-maker pairs, A or B, in a 2AFC. The dependent variable was the correct rate informed by the proportion of participants who chose the matching pair group out of the total number of participants. The remaining procedures were the same as those used in Experiment 1.

### 3.2. Results and discussions

The rate of participants choosing the correct pairs was 65.96% (62 participants chose the matching pairs while 32 participants chose the mismatching pairs); this, indicating a significant difference [*χ*^2^ (1) = 9.57, *φ* = 0.32, *p* < 0.01]. The results demonstrate that people can match dolls to their makers, even if only the back views of dolls are presented.

These results may indicate that people can match dolls and their makers based on information other than faces. Thus, there are two possibilities. First, people might match dolls and their makers based on their physical traits (e.g., body shape, hair, colors). However, the features common to the front views of the students and the back views of the dolls were limited. Second, the matching might have occurred based on other information. For example, couples resemble in their psychological and personality traits (Keller et al., 1996; Little et al., 2006; Wong et al., 2018), suggesting that dolls and their makers may resemble in their perceived personality traits.

Experiment 3 examined the second possibility, namely, that psychological traits affected matching decisions. Specifically, if the matched (correct) pairs were judged to resemble psychological traits more, it would suggest that the matching judgment in Experiment 2 was not based on physical traits other than the faces, but psychological traits. Conversely, if the matched pairs were judged to resemble fewer psychological traits, the results of Experiment 2 could be interpreted as judging based on non-facial physical traits.

## 4. Experiment 3

### 4.1. Experiment 3A

Experiment 3 examined whether people could match dolls to their makers based on personality traits. Of course, the doll itself should have no personality traits, but the makers may project themselves when making the dolls. For example, people might perform a doll-maker matching task based on the following guesses: “She looks kind. So, she may make dolls that have a kind attitude” or “This doll looks naive. The maker may have also been naive.” To investigate this possibility, we examined the correlations between ratings of the personalities of the dolls and students in Experiment 3A.

If the matching judgment is based on personality, the positive correlations should be the appropriate clues, because it means that the personalities of dolls and their makers are similar. However, negative correlations should be false or bad clues because the personalities do not resemble each other. Therefore, if the personality traits affect matching judgments, more significant positive correlations would be obtained between the personality traits of the matching doll-maker pairs than those of the mismatching pairs. On the contrary, if personality traits do not affect matching judgments, the matching pairs will not yield more positive correlations than the mismatching pairs.

#### 4.1.1. Methods

##### 4.1.1.1. Participants

Thirty-one female college students in Tokyo voluntarily participated in this experiment. Data from one participant were eliminated from the analysis because of missing values. Fifteen participants (average 19.00 ± 0.85 years old) rated the personalities of the front views of the dolls and students, the other 15 participants (average 19.29 ± 1.10 years old) rated those of the back views of dolls and the front views of students. The sample size was determined based on power analysis [the calculated sample size was 17, with effect size = 0.6, *α* error probability = 0.05, and power (1 − *β* error probability) = 0.8].

##### 4.1.1.2. Stimuli

Photos of eight dolls and their makers used in Experiment 2 were also used in this experiment as matching doll-maker pairs (Pairs 1–8). We also intended to use the photos of eight dolls and students consisting of mismatched pairs used in Experiment 2; accidentally, we used two other students’ photos. Thus, the two students’ photos that were not used in Experiment 2 but used in Experiment 1 were used in this Experiment 3A. Eight mismatched pairs were randomly determined (Pairs 9–16).

There were two types of questionnaires: one presented the front views of the dolls (Figure 1C) and students (Figure 1A), and the other presented the back views of the dolls (Figure 1E) and the front views of the students (Figure 1A). Each questionnaire contained 16 photos of each doll and student; each A4 questionnaire sheet presented two to three photos of the dolls or students and 10 questions about the personality (described later) next to each photo. The first and second halves of the questionnaire were about the students and dolls, respectively, for judging their personality traits. The students’ and dolls’ photos were about 3.5 cm × 3.5 cm, and 2.0 cm × 3.0 cm, respectively.

We used TIPI−J (Oshio et al., 2012) to capture the characteristics of personality of dolls and students with 10 questions. TIPI−J (Oshio et al., 2012) was standardized in Japanese based on the 10-Item Personality Inventory (TIPI; Gosling et al., 2003). TIPI (Gosling et al., 2003) was based on the Big Five model, which captures personality with the five factors of extraversion, agreeableness, conscientiousness, emotional stability, and openness to experience.

##### 4.1.1.3. Procedures

One questionnaire was randomly distributed among the participants. We asked the participants to rate the personalities of the dolls and students using the questionnaire. When the participants mentioned that dolls did not have personalities (few people did so), we asked them to rate the personalities of the dolls’ makers. They then answered 10 personality questions on a seven-point Likert scale for each photo. There was a total of 320 questions (10 personality questions × 32 photos (16 dolls +16 students)); the task took about 20 min, including instructions. The remaining procedures were the same as those used in Experiment 1.

#### 4.1.2. Results and discussions

Ratings of the five personality traits were obtained in this experiment. Kendall’s rank correlation coefficients between the dolls and their makers were calculated for each pair. Table 1 shows all significant correlations obtained with the matching and mismatching pairs. Overall, more positive than negative correlation coefficients were obtained for the matched pairs.

**Table 1:** Significant correlations shown in Experiment 3A and pairs used in Experiment 3B.

		Front (dolls)—Front (student)		Back (dolls)—Front (students)	
		Significant correlation	Clue in Experiment 3B	Significant correlation	Clue in Experiment 3B
Matching pairs (correct pairs)	Pair 1		Neutral		−
	Pair 2	**Extraversion** (*τ* = 0.473)^*^Agreeableness (*τ* = 0.620)^**^	Appropriate		−
	Pair 3	*Agreeableness* (*τ* = −0.447)^*^	False		Neutral
	Pair 4		Neutral		Neutral
	Pair 5		−		False
	Pair 6	**Conscientiousness** (*τ* = 0.575)^*^	Appropriate	*Agreeableness* (*τ* = −0.453)^*^	False
	Pair 7		False	**Emotional Stability** (*τ* = 0.644)^**^	Appropriate
	Pair 8	**Conscientiousness** (*τ* = 0.553)^*^	−	**Conscientiousness** (*τ* = 0.513)^*^	Appropriate
Mismatching pairs (incorrect pairs)	Pair 9		−		−
	Pair 10	*Emotional Stability* (*τ* = −0.500)^*^	Appropriate	**Agreeableness** (*τ* = 0.560)^*^	False
	Pair 11	**Conscientiousness** (*τ* = 0.470)^*^	False		False
	Pair 12		−		Neutral
	Pair 13	**Conscientiousness** (*τ* = 0.494)^*^	False		Neutral
	Pair 14		Neutral		Appropriate
	Pair 15		Neutral		−
	Pair 16	*Openness to Experience* (*τ* = −0.472)^*^	Appropriate	*Agreeableness* (*τ* = −0.527)^*^	Appropriate

**Bold** means positive correlation, and *Italic* means negative correlations.

* *p* < 0.05;

** *p* < 0.01.

Contrary to our predictions, both significantly positive and negative correlations were also obtained as shown in Table 1. However, this would not matter because it is assumed that not all doll-maker pairs would be equally used for judgments. The questionnaire used in Experiments 1 and 2 consisted of multiple pairs, and the participants chose the matching-pair group. In this case, if some of the multiple pairs are strongly similar, the participants could make a correct judgment based on them. For example, it would be possible for the participants to follow strategies such as “Personality traits of Pair 2, Pair 6, and Pair 8 are so similar. The group including these pairs could be matching doll-maker pairs.” That is, not all matching pairs are expected to have consistently strong and positive correlations.

The results of Experiment 3A show that the personality traits of matching doll-maker pairs were more similar than those of mismatching pairs. However, some positive correlations were observed even for the mismatched pairs. Therefore, it may be difficult to conclude whether the correct pairs resemble psychological traits more and whether personality traits affect matching judgments. We then examined whether the resemblance of personality traits affected judgment in Experiment 3B.

### 4.2. Experiment 3B

In Experiment 3B, we extracted the photos of pairs as appropriate, neutral, and false clues based on the correlations between the dolls and students obtained in Experiment 3A to examine whether the degree of resemblance in personality traits affects judgment. If the matching judgment is based on personality, the positive correlations in matching pairs may be appropriate clues, because it means that the personalities of the dolls and their makers are similar. Also, the negative correlations in the matching pairs may be false or bad clues because the personalities do not resemble each other. However, the negative correlations of their personality traits could be appropriate clues, and the positive correlations could be negative clues in mismatched pairs for matching judgments. Pairs that did not show significant correlations were neutral clues for matching judgment in both matching and mismatching pairs. If the personality affects the doll-maker judgment, the correct rates may be high, moderate, and low under appropriate, neutral, false clue conditions, respectively. We tested these predictions by using three types of clues in Experiment 3B.

#### 4.2.1. Methods

##### 4.2.1.1. Participants

Thirty-five female college students (average 19.74 ± 1.25 years old) and 28 university students (13 females and 15 males; average 19.68 ± 0.60 years old) in Tokyo voluntarily participated in this experiment. The sample size was determined based on power analysis [the calculated sample size was 32, with effect size (*w*) = 0.5, *α* error probability = 0.05, power (1 − *β* error probability) = 0.8, and *df* = 1; we assumed a larger effect size than in Experiment 1 because it was expected to have higher correct rates than in Experiments 1 and 2 under the appropriate clue condition]. Thirty-two of them participated *via* a questionnaire to judge the matching pairs of the front views of dolls, and 31 participated to judge the matching pairs of the back views of dolls.

##### 4.2.1.2. Stimuli

We extracted two matching pairs and two mismatching pairs each for appropriate, neutral, and false clues from the pairs used in Experiment 3A, based on their correlations. “Clue in Experiment 3B” in Table 1 indicates which pairs were used as appropriate, neutral, or false clues. The clues were reflected implicitly in the photographs. The only stimulus in Experiment 3B was a photograph, with no personality rating or no description of which photographs were appropriate, neutral, or false conditions. We used photos of 12 pairs as stimuli for front and back views of the dolls (note that pairs that show no significant correlations were also used as appropriate or false clues because we did not have enough pairs with significant positive or negative correlations. Pairs with no significant correlations were randomly assigned to each clue condition). Some pairs were not used to equalize the number of pairs in the three clue conditions.

Two types of questionnaires were prepared. One asked about the resemblance between the front views of the dolls (Figure 1C) and those of the students (Figure 1A), and the other asked about the resemblance between the back views of the dolls (Figure 1E) and the front views of the students (Figure 1A). Each questionnaire consisted of three questions corresponding to three clue conditions (i.e., an appropriate clue, a neutral clue, and a false condition), which had two matching and two mismatching pairs. For example, an appropriate clue condition for the front views of dolls consists of two matching pairs (Pairs 2 and 6) and two mismatching pairs (Pairs 10 and 16). Squares with different colors surrounded each matching and mismatching group, and were labeled A or B. Each question was printed on a separate sheet. Stimulus photos were allocated to 2 × 2 in each group. The students’ and dolls’ photos were about 3.6 cm × 3.6 cm, and 2.5 cm × 3.5 cm, respectively. All the photos were printed in color. The positions of the matching pairs and the order of the questions were changed to yield 12 questionnaire patterns.

##### 4.2.1.3. Procedures

One questionnaire was given to each participant who answered three questions on the front or back views of the dolls. Participants looked only at the photographs of the doll-student pairs and were asked to choose pairs in which dolls and students resembled each other in a 2AFC, answering three questions in total. The test took approximately 5 min including instructions. The dependent variable was the proportion of participants who chose the matched pair group out of the total number of participants. The remaining procedures were the same as those used in Experiment 1.

#### 4.2.2. Results and discussions

Table 2 shows the rates at which the matching pairs were chosen and the results of the *χ*^2^ tests. Regarding to the front views of dolls, the rate of choosing the matching pairs under the neutral clue condition was the highest, and the rate under the appropriate clue condition was the lowest, contrary to our expectations. However, the rates were highest in the appropriate clue condition, followed by the neutral clue condition, and lowest in the false clue condition for back views of dolls; this concurs with our predictions. The number of choosing matching pairs was significantly larger than that of mismatching pairs under the appropriate clue of the back views condition. Experiment 2 found that people could match dolls to their makers even with the back views of dolls. Thus, this could indicate that people tend to match dolls to their makers based on their resemblance of personalities when the dolls’ faces are invisible. However, this tendency was not observed in the front-view conditions, demonstrating that people match dolls to their makers based on resemblance, such as appearance when dolls’ faces are visible but based on the personality when dolls’ faces are invisible.

**Table 2:** The numbers and rates of choosing the matching pairs.

View of dolls	Clue	Rate of correct pair choice	Number of correct pair choices	Number of incorrect pair choices	Chi-square tests (two-sided)
Front	Appropriate	37.50%	12	20	*χ*^2^ (1) = 2.00, *ϕ* = 0.25, *n.s.*
	Neutral	75.00%	24^*^	8^*^	*χ*^2^ (1) = 8.00, *ϕ* = 0.50, *p* < 0.01
	False	46.88%	15	17	*χ*^2^ (1) = 0.13, *ϕ* = 0.06, *n.s.*
Back	Appropriate	70.97%	22^*^	9^*^	*χ*^2^ (1) = 5.45, *ϕ* = 42, *p* < 0.05
	Neutral	61.29%	19	12	*χ*^2^ (1) = 1.58, *ϕ* = 0.23, *n.s.*
	False	35.48%	11	20	*χ*^2^ (1) = 2.61, *ϕ* = 0.29, *n.s.*

* Significantly high or low according to residual analysis.

## 5. General discussions

Uetsuki and Kimura (2022) demonstrated a resemblance between the dolls and their makers; however, the reason for this was not apparent. In this study, we examined which part of the dolls resembled their makers. Experiments 1 showed that people could match the dolls with their makers when they lacked eye regions. Experiment 2 also demonstrated that people could match dolls to their makers above a chance level based on the back views of dolls. Experiment 3 examined the possibility that the makers project their personality traits onto dolls, and that people match the dolls with their makers based on their personality. Experiment 3B examined whether the degree of similarity in personality traits obtained in Experiment 3A affected the judgment of resemblance between the dolls and their makers. The results showed that people judged the matching doll (back view)–maker (front view) pairs whose rated personalities had positive correlations as more similar. This result indicates that people match dolls to their makers based on resemblances other than appearance, such as personality traits, when dolls’ faces are invisible.

In contrast, people did not judge the matching doll (front view)–maker (front view) pairs whose rated personalities had positive correlations as more similar. The difference in the results between the front and back views may suggest that people use resemblance of appearance to make a matching judgment when they can get much information about appearance, but people use resemblance of personality traits to make a matching judgment when they cannot get much information about appearance. This raises the question of how people match dolls with their makers when dolls’ faces are visible? Multiple elements that make up the face (i.e., eyes, eyebrows, nose, and mouth), their arrangement, or face lines might affect matching judgment. Therefore, future studies are required.

This study demonstrated that doll-maker matching is not based merely on the eye region, in contrast to dog-owner matching (Nakajima, 2013). These findings suggest that the resemblances in psychological traits might be also observed between dogs and their owners. We also demonstrated that matching was possible, even with the back views of the dolls. Additionally, we showed that the resemblance of personality traits assumed by the dolls and their makers influences matching judgment when dolls’ faces are invisible. However, this study has two limitations. First, Experiment 3 had a small number of stimuli, and a limited number of matched and mismatched pairs were used. Second, we cannot explain why dolls resemble their makers. Although this study demonstrated that people project themselves not only when selecting but also when making something, it does not clarify the mechanism by which the makers projects themselves onto dolls. This requires further investigation.

People project themselves both while selecting and making something (Uetsuki and Kimura, 2022). Furthermore, the current study showed that people project both their physical and psychological traits into their works even if they are amateur. This study may also provide evidence for the validity of drawing tests that measures personality, such as the house-tree-person (HTP) test (Buck, 1948) that is used often in clinical psychology. Furthermore, this study suggests that various information including psychological traits can be obtained from works. Despite the passing of the creators of famous Buddha statues, paintings, or manga, it could be possible to capture the psychological traits of that person from his or her works. Thus, this study may contribute to the multiphase understanding of creators by capturing them from their works as well.

## Data availability statement

The datasets presented in this study can be found in online repositories. The names of the repository/repositories and accession number(s) can be found in the article/Supplementary material.

## Ethics statement

The studies involving human participants were reviewed and approved by the Ethics Committee of Hakodate Junior College, or of the Ethics Regulations Concerning Research Involving Human Subjects of Aoyama Gakuin University. The patients/participants provided their written informed consent to participate in this study. Written informed consent was obtained from the individuals for the publication of any identifiable images included in this article.

## Author contributions

MU and MK conceived, designed, performed the experiments at Hakodate Junior College, Aoyama Gakuin Women’s Junior College, and Aoyama Gakuin University, and analyzed the data. MU wrote up the study. All authors contributed to the article and approved the submitted version.

## Funding

This work was supported by JSPS KAKENHI grant number JP16K12516 to MK and MU. Open access publication fees will be received from Aoyama Gakuin University.

## Conflict of interest

The authors declare that the research was conducted in the absence of any commercial or financial relationships that could be construed as a potential conflict of interest.

## Publisher’s note

All claims expressed in this article are solely those of the authors and do not necessarily represent those of their affiliated organizations, or those of the publisher, the editors and the reviewers. Any product that may be evaluated in this article, or claim that may be made by its manufacturer, is not guaranteed or endorsed by the publisher.
